# Nourseothricin as a novel drug for selection of transgenic *Giardia lamblia*

**DOI:** 10.1016/j.ijpddr.2024.100543

**Published:** 2024-04-26

**Authors:** Corina D. Wirdnam, Dawid Warmus, Carmen Faso

**Affiliations:** a Institute of Cell Biology, University of Bern, Baltzerstrasse 4, 3006 Bern, Switzerland; b Graduate School for Cellular and Biomedical Sciences, University of Bern, Mittelstrasse 43, 3012 Bern, Switzerland; c Multidisciplinary Center for Infectious Diseases, University of Bern, Hallerstrasse 6, 3012 Bern, Switzerland; d Institute for Infectious Diseases, University of Bern, Friedbuehlstrasse 25, 3001 Bern, Switzerland

**Keywords:** Antibiotic, Resistance, Selection marker, *Giardia lamblia*, Transgenesis, Transfection, Drug

## Abstract

Functional gene and protein characterizations in parasitic protists are often limited by their genetic tractability. Despite the development of CRISPR-Cas9-derived or inspired approaches for a handful of protist parasites, the overall genetic tractability of these organisms remains limited. The intestinal parasite *Giardia lamblia* is one such species, with the added challenge of a paucity of reliable selection markers.

To address this limitation, we tested the feasibility of using Nourseothricin as an effective selection agent in Giardia. Here, we report that axenically-grown WB *Giardia* cells are sensitive to Nourseothricin and that engineering expression of the streptothricin acetyltransferase (SAT-1) gene from *Streptomyces rochei* in transgenic parasites confers resistance to this antibiotic. Furthermore, we determine that SAT-1-expressing parasites are cross-resistant neither to Neomycin nor Puromycin, which are widely used to select for transgenic parasites. Consequently, we show that Nourseothricin can be used in sequential combination with both Neomycin and Puromycin to select for dual transfection events.

This work increases the number of reliable selection agents and markers for *Giardia* genetic manipulation, expanding the limited molecular toolbox for this species of global medical importance.

## Graphical abstract

Created with BioRender.com.

**Figure:**
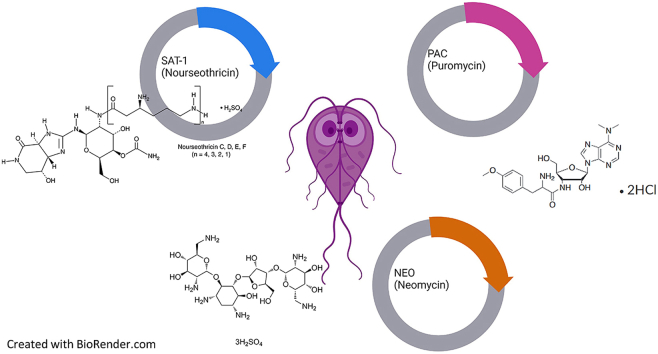

## 1 Introduction

Parasitic protists have garnered attention as novel model organisms for cell and molecular biology investigations focused on cell and organelle evolution (Dacks and Ginger, 2023). Beyond their global medical relevance, parasitic protists often present surprising and extreme variations of the eukaryotic cell “blueprint”, with profound modifications and/or loss of entire organelle systems (Faso et al., 2013), secondary and even tertiary endosymbiotic events unknown in well-characterized model organisms (Boucher and Yeh, 2019), and unique gene regulation mechanisms (Romagnoli et al., 2020). Genetic tractability of parasitic protists is a pre-requisite for functional characterizations in these species. The first step towards complete genetic tractability is the ability to generate genetically modified i.e., transgenic lines for further investigation.

The protist parasite *Giardia lamblia (syn. Giardia duodenalis* and *Giardia intestinalis)*, causative agent of the diarrheal disease Giardiasis, presents a severely reduced organellar composition and is therefore a useful model cell to investigate basic biological mechanisms such as intracellular and secretory protein trafficking (Ankarklev et al., 2010; Cernikova et al., 2018; Faso and Hehl, 2019) as well as a range of eukaryotic biology such as genomic minimalism, cell differentiation and bacterial-like metabolic enzymes (Han and Collins, 2012; Ihara et al., 2022; Morrison et al., 2007; Svärd et al., 2003).

Since the 1980s and despite its overall low genetic tractability, *G. lamblia* has been the object of intense cell and molecular biology investigations. Recent advances in adapting variants of the CRISPR-Cas9 tool have shown that it is possible to use this approach to generate transgenic lines with lowered levels of expression of a selected target gene (Horáčková et al., 2022; Jex et al., 2020; McInally et al., 2019). However, the paucity of effective selection agents for *in vitro G. lamblia* studies remains a major limitation for extensive genetic manipulations requiring multiple genomic insertions such as generation of gene knock-out lines (Ebneter et al., 2016; Wampfler et al., 2014), with Neomycin (NM) and Puromycin (PM) the only reliable drugs for selection to date, and Blasticidin a poor alternative (Ebneter et al., 2016).

Nourseothricin (NT) is a member of the streptothricin-class of aminoglycoside antibiotics produced by Streptomyces species and has been used as a selection marker in a wide range of organisms including bacteria, yeast, filamentous fungi, plant cells, and parasitic protists such as Leishmania and Trypanosoma (Freedman and Beverley, 1993; Joshi et al., 1995; Knüsel et al., 2022; Stellner et al., 2023; Zhu et al., 2024). Resistance to NT is conferred by expression of the streptothricin acetyltransferase (SAT-1) gene from *Streptomyces rochei*.

In this report, we show that NT is an effective drug for the selection of transfected *G. lamblia* lines and that expression of the *S. rochei* SAT-1 gene in transgenic *Giardia* cells confers resistance to this antibiotic, with no detectable cross-resistance either to NM or PM. In line with this, we show that NT can be used alone or in sequential combination with both NM and PM to select for dual transfection events. This technical advance contributes to the much-needed expansion of the genetic toolbox for the full gamut of genetic manipulations in *Giardia*, increasing the genetic tractability of this globally relevant parasite.

## 2 Materials and Methods

### 2.1 Cell culture, selection of transgenic lines and cell counting

*G. lamblia* trophozoites were cultured in well-established described axenic conditions (Ebneter et al., 2016; Faso et al., 2013; Wampfler et al., 2014). WB cells (Smith PD et al., 1982) were transfected with circular or SwaI-linearized plasmids following the protocols previously published (Yee and Nash, 1995). Briefly, cells from confluent cultures were detached on ice centrifuged at 900 g for 10 min and resuspended in fresh medium to a concentration of ca. 30 million cells/ml. An aliquot containing *ca.* 10 million cells was mixed with 50 μg DNA and electroporated in a 0.4 cm-gap cuvette at 350 V, 1000, μF, and 720Ω, followed by incubation on ice for 15 min and resuspension in fresh medium. The antibiotic selection was started 24 h after electroporation, using PM (36 μg/ml; Invivogen-ant-pr-1), NM (410 μg/ml; Sigma G8168-10 ML) or NT (1 mg/ml; Jena Bioscience-AB-102 L). Cell counting was performed using a Neubauer chamber.

### 2.2 Measuring cell culture occupancy with image segmentation

For the measurement of cell occupancy, 10′000 cells/well were inoculated in a 96-well plate [Greiner Cellstar 655090] with the addition of NT, PM or NM. Cells were incubated in an oxygen-depleting pouch [Anaerogen Z-compact, ThermoFisher AN0010W]. Images were taken in wide-field with Eclipse Ti2-E (inverted microscope) connected to a high-throughput imaging platform (LIPSI) [Nikon]. For each well, at least ten images were taken randomly and segmented into binary masks using the University of Bern high-power computing system UBELIX with the open-source Python library Cellpose and pre-trained model “cyto”. Objects in masks were filtered based on pixel area and analysed with the Python library skimage.

### 2.3 Luciferase activity measurement

Equal volumes of detached confluent cultures were analysed using Nano-Glo® Luciferase Assay [Promega #N1110] and the reader Centro XS³ LB 960 Microplate Luminometer [Berthold Technologies], using the software MikroWin 2010 version 5.15 (Mikrotek Laborsysteme GmbH, Germany).

### 2.4 Plasmid construction

Constructs were generated using standard recombinant DNA and cloning techniques. All the details about the used templates, primers, vectors and restriction enzymes are found in Table 1.

**Table 1:** List of constructs presented in this report, including those acquired and newly-synthesized.

Plasmid	Template for insert	Primers	Backbone vector	Enzymes
P7	genomic DNA of *G. lamblia* WB clone 6 (Smith PD et al., 1982)	CF9 (gcTCTAGATGAACCTGACCCTAGGAGCTTTTC) + CF10 (gcTTAATTAActaCGCGTAGTCTGGGACATCGTATGGGTACTCCATCTTGCAGTCATGCAAGAAG)	P6- a derivation of pPACV-Integ (Jiménez-García et al., 2008; Singer et al., 1998)	XbaI and PacI
P8	pNL1.1- [Nluc] Vector (Promega)	CF12 (gactATGCATGTCTTCACACTCGAAGATTTCGTTG) + CF2 (gcTTAATTAActaCGCGTAGTCTGGGACATCGTATGGGTACGCCAGAATGCGTTCGCACAG)	P5- a derivation of RAN-Neo vector (Hehl et al., 2000; Sun et al., 1998a)	NsiI and PacI
P10	pHTC HaloTag CMV-neo Vector (Promega)	CF13 (gactATGCATGGATCCGAAATCGGTACTGGC) + CF4 (gcTTAATTAActaCGCGTAGTCTGGGACATCGTATGGGTAACCGGAAATCTCCAGAGTAGAC)	P5	NsiI and PacI
P11	P8	CF7 (GACAACTTTTTCTGTAAACGTG) + CF15 (gcTTAATTAActaCGCCAGAATGCGTTCGCACAG)	P6	XbaI and PacI
P13	P10 digested with XbaI and PacI	–	P6	XbaI and PacI
P14	pLEX SAT (Joshi et al., 1995)	CF22 (gactATGCATATGAAGATTTCGGTGATCCCTGAG) + CF23 (gcTTAATTAActaGGCGTCATCCTGTGCTCCCG)	P5	NsiI and PacI
P15	P14 digested with XbaI and PacI	–	P6	XbaI and PacI
P62	pLEX SAT for step 1, P7 for step 2. In step 3 a mix of the products from step 1 and 2 was used as template	CF105 (catGATATCGAAGCGCTGACCACAAATAACG) + CF106 (AGGTAAATATTCACTTCAGCCCCTAGGCGTCATCCTGTGCTCCC) for step 1, CF107 (GGGAGCACAGGATGACGCCTAGGGGCTGAAGTGAATATTTACCT) + CF108 (gcTCTAGAGAATTCGAGCTCGGTACC) for step 2, CF105 + CF108 for step 3	P7	EcoRV and XbaI
P79	P11 digested with XbaI and PacI	–	P62	XbaI and PacI

### 2.5 Immunoblotting analyses for protein expression in *Giardia lamblia*

Equal numbers of cells (*ca.* 2 × 10^6^) from detached confluent cultures were subjected to standard immunoblotting analysis using the primary anti-HA epitope antibody [Sigma #11867431001] and the secondary anti-rat antibody coupled to HRP [Sigma #A9037-1 ML]. Membranes were developed with the Pierce™ ECL Plus Western Blotting Substrate [ThermoFisher #32132].

## 3 Results

### 3.1 Nourseothricin inhibits growth of *in-vitro* cultured non-transfected *Giardia lamblia* cells

**Fig. 1:**
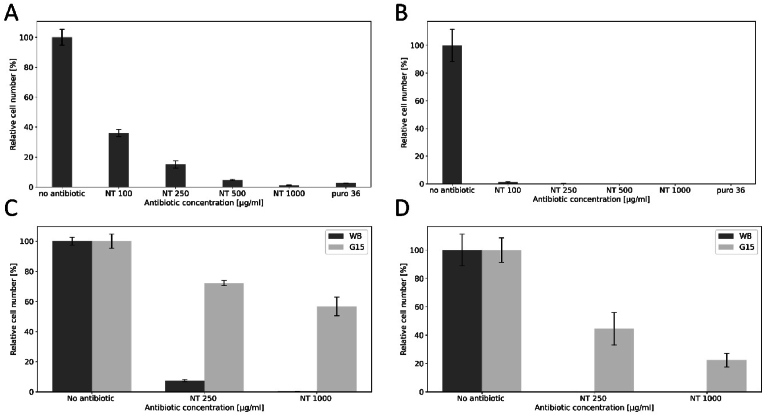
**Effect of NT and PM on WB and transgenic Giardia line G15.** Effect of NT and PM on WB (dark grey) cells after 1 or 2 days' treatment (A, B). Effect of two NT concentrations on WB (dark grey) and G15 (light grey) cells, after 1 or 2 days' treatment (C, D). Briefly, on day 0, confluent cultures were detached on ice and fresh medium was inoculated at a dilution 1:10 for all antibiotic concentrations. After 1 day, all cultures were detached on ice, aliquots were used for cell counting and fresh medium was inoculated again at a dilution 1:10 irrespective of the cell number. After one more day, cells were detached and counted. In each graph, the control culture containing no antibiotic was taken as reference for the calculation of % reduction due to antibiotic treatment.

To determine whether NT can be used as an effective selection agent for *G. lamblia* transfection, the first step was to test sensitivity of in-vitro cultured *G. lamblia* cells to the drug. To do this, we performed preliminary experiments (data not shown) with an approximate testing range of NT concentrations based on what we routinely use for selection with PM (36 μg/ml). However, we soon realized that WB cell growth inhibition, with similar dynamics to PM treatment, could only be achieved using significantly higher NT concentrations. Thus, we tested growth of WB *G. lamblia* cells in the presence of 100, 250, 500 and 1000 μg/ml NT or 36 μg/ml PM, over a period of 48 h, including an untreated control. The data in Fig. 1A and B clearly show how NT can effectively inhibit growth *G. lamblia* cells, albeit less efficiently than PM.

### 3.2 *S. rochei* streptothricin acetyltransferase expression in transgenic *Giardia lamblia* cells confers resistance to nourseothricin

Having determined that WB *G. lamblia* cells are inhibited by exposure to NT, we next sought to engineer resistance to this antibiotic. To do this, we tested the effect of expression in *G. lamblia* of the gene coding for streptothricin acetyltransferase (*SAT-1*), which had been previously used to confer NT resistance in Leishmania cells (Joshi et al., 1995). We created construct P15, consisting of the SAT-1 ORF amplified from plasmid pLEX SAT (Joshi et al., 1995) and cloned in plasmid pPACV-Integ (Singer et al., 1998) downstream of the derived promoter and 5′UTR regulatory sequences of the constitutively expressed GDH gene (Wampfler et al., 2014). Following episomal transfection with P15 and selection of the corresponding *G. lamblia* line G15 using PM, transgenic *G. lamblia* cells were challenged with 250 and 1000 μg/ml NT in the absence of PM and alongside non-transfected cells, for 48hrs. The data for cell numbers recorded in Fig. 1C and D shows that, with respect to WB cells, line G15 grows in the presence of NT.

### 3.3 *S. rochei* streptothricin acetyltransferase is an independent selectable marker for transfection of *Giardia lamblia* cells

**Fig. 2:**
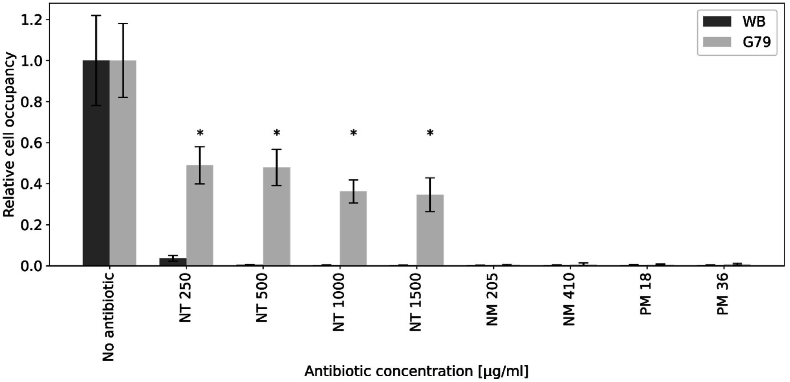
**Cell occupancy of WB and G79 Cell Lines in response to antibiotic treatment.** WB (dark grey bars) and G79 (light grey bars) transgenic cells were tested for growth in the absence or in the presence of the indicated NT, NM and PM concentrations. Growth was measured in terms of relative cell occupancy with respect to untreated cells. *: statistically significant difference in cell occupancy (One-Way ANOVA, p-value is < 0.00001). Images were acquired in pre-defined settings using the LIPSI high-throughput imaging platform, and calculations were made as described in “Materials and Methods”.

We demonstrated that transfection of the SAT-1 cassette in *G. lamblia* cells prevented NT-mediated growth inhibition. To test whether SAT-1 expression would confer resistance exclusively to NT, we constructed a variant of plasmid pPACV-Integ called pSATV-Integ (plasmid P79), in which the PAC expression cassette conferring resistance to PM is substituted with the SAT-1 expression cassette previously tested in P15. As in pPACV-Integ, the SAT-1 cassette in pSATV-Integ is flanked by regions of homology adjacent to the TPI locus (Lu et al., 2002), allowing for its stable insertion in the genome by homologous recombination (Singer et al., 1998; Sun et al., 1998b). In addition, pSATV-Integ carries an NLuc expression cassette, under the control of a derived GDH promoter. *G. lamblia* cells were stably transfected with pSATV-Integ to generate line G79, and selected using 1 mg/ml NT. After selection, NT treatment was suspended and, following 48hrs exposure to 205 and 410 μg/ml NM or 18 and 36 μg/ml PM, both WB and G79 cell numbers were counted. The data recorded in Fig. 2 show how line G79 survived and grew in the presence of NT (statistically significant difference in cell occupancy, p-value is < 0.00001 by one-way ANOVA testing).

### 3.4 Nourseothricin, neomycin and puromycin can be used in combination for dual transfection experiments

**Fig. 3:**
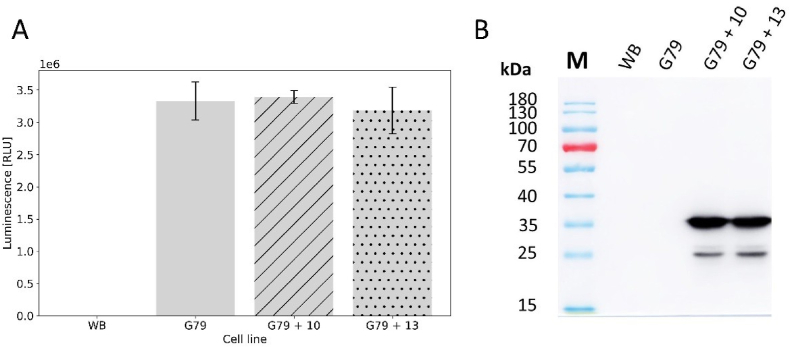
**Dual selection following supratransfection, and analysis of transgene expression.** (A) Luciferase activity of WB and transgenic Giardia Lines G79, G79 + 10 and G79 + 13. Luminescence (in Relative Light Units, RLU) was measured for equal numbers (*ca.* 2 × 10^6^) of WB and G79 cells, along with supratransfected G79 + 10 and G79 + 13 cells following selection with NM and PM, respectively. (B) Epitope-tagged HaloTag gene expression analysed by immunoblotting. Protein extracts from equal cell numbers (*ca.* 2 × 10^6^) of Giardia cell lines WB, G79, G79 + 10, and G79 + 13 were prepared with protein sample buffer, subjected to PAGE and probed with an anti-HA antibody in immunoblotting experiments.

Having determined that resistance to NT by expression of the SAT-1 cassette confers cross-resistance neither to NM nor to PM, we validated this observation by transfecting line G79 with plasmids P10 and P13, which both carry an epitope-tagged HaloTag expression cassette (Los et al., 2008), selectable using NM and PM, respectively. Following supra-transfection of G79 cells with either plasmid P10 (G79 + 10) or P13 (G79 + 13) and selection with 410 μg/ml NM and 36 μg/ml PM, respectively, we measured comparable NanoLuc expression in all transgenic lines (Fig. 3A), consistent with the presence of a stably integrated NanoLuc expression cassette in the parental line G79. Clear detection of the epitope-tagged HALO reporter by immunoblotting demonstrated that NT can be used in combination with either NM or PM for sequential transfection and selection experiments (Fig. 3B).

## 4 Discussion

Metagenomics approaches have made it possible to sample and investigate the incredible diversity and evolutionary history of eukaryotic life, with a special focus on both free-living and parasitic protists of incalculable ecological and medical relevance (Hempel et al., 2022; Wylezich et al., 2020). However, to move beyond a collection of genomic sequences towards functional characterization of protein complexes and molecular machineries, it is essential that tools for transgenesis and other genetic manipulations be also developed. Indeed, the availability of robust genetic manipulation tools is the first hurdle in the establishment of any organism as an experimental model.

Molecular and cell biology investigations in *G. lamblia* have often been hindered by a lack of robust genetic manipulation tools, with the added challenge of *G. lamblia*'s tetraploid status and lack of a sexual cycle. Morpholinos initially held great promise (Carpenter and Cande, 2009), as did the discovery of an RNAi pathway in this species (Rivero et al., 2010). Electroporation-based transfection protocols for *G. lamblia* were developed early on, including transduction approaches using *G. lamblia* virus GLV (Yu et al., 1995). Robust selection of transgenic cell populations is the first and most fundamental step towards functional investigations in any organism. In *G. lamblia*, only two drugs are currently considered robust selection agents, neomycin and puromycin.

In this report, we provide proof-of-concept for NT as a useful and robust selection agent. We show that the SAT-1 gene can be effectively expressed in *G. lamblia* cells to confer resistance uniquely to NT, with no observed cross-resistance to either NM or PM. This is a significant contribution to the *G. lamblia* toolbox in that three selection systems can now be used.

In our experiments, we employed sequential selection with NT and NM or PM as the most parsimonious approach towards testing the NT/SAT-1 system. However, it is likely that the three antibiotics can be used simultaneously, allowing for a triple transfection and selection procedure of episomally-maintained constructs. To minimize antibiotic usage while ensuring stable levels of transgene expression, it would be advantageous to exchange the homologous recombination regions flanking the SAT-1 cassette in pSATV-Integ with regions for integration in another genomic site. This would ensure that both pPACV-Integ and pSATV-Integ can be stably integrated in the genome, with no need for drug selection.

An important consequence of simultaneous selection using three drugs is the potential for engineering the concomitant disruption/substitution of three out of four alleles in the *G. lamblia* genome, a very time-consuming process previously performed using just NM and PM in a series of sequential homologous recombination-based gene disruptions, followed by Cre-*lox*P-mediated “recycling” of the selection marker (Ebneter et al., 2016; Wampfler et al., 2014). With three markers, it should be possible to limit selection marker recovery to just one gene, without the need to select for Cre recombinase expression, given that only a handful of Cre molecules are needed to excise a “floxed” fragment. The recovered selection marker could then be used to generate a full knock-out line, with 4/4 disrupted alleles. Furthermore, this approach would also allow for rigorous mutagenesis investigations by performing the characterization of mutant proteins in a “clean” genetic background, in the absence of any wild-type molecules which might mask a phenotype of interest.

## CRediT authorship contribution statement

**Corina D. Wirdnam:** Conceptualization, Data curation, Formal analysis, Investigation, Methodology, Writing – original draft, Writing – review & editing. **Dawid Warmus:** Data curation, Formal analysis, Investigation, Methodology, Software, Writing – original draft, Writing – review & editing. **Carmen Faso:** Conceptualization, Funding acquisition, Methodology, Project administration, Resources, Supervision, Visualization, Writing – original draft, Writing – review & editing.

## Declaration of competing interest

The authors declare no conflicts of interest.
