# Supplementary material for: Nourseothricin as a novel drug for selection of transgenic Giardia lamblia
Source: Int J Parasitol Drugs Drug Resist. 2024 Apr 26;25:100543. doi: 10.1016/j.ijpddr.2024.100543 (PMC11067369; doi:10.1016/j.ijpddr.2024.100543)
Supplement: Multimedia component 4 [file mmc4.docx]

Well from 96 well plate

Randomized 10-15 images per well

2048 X 2048 px

Cellpose analysis


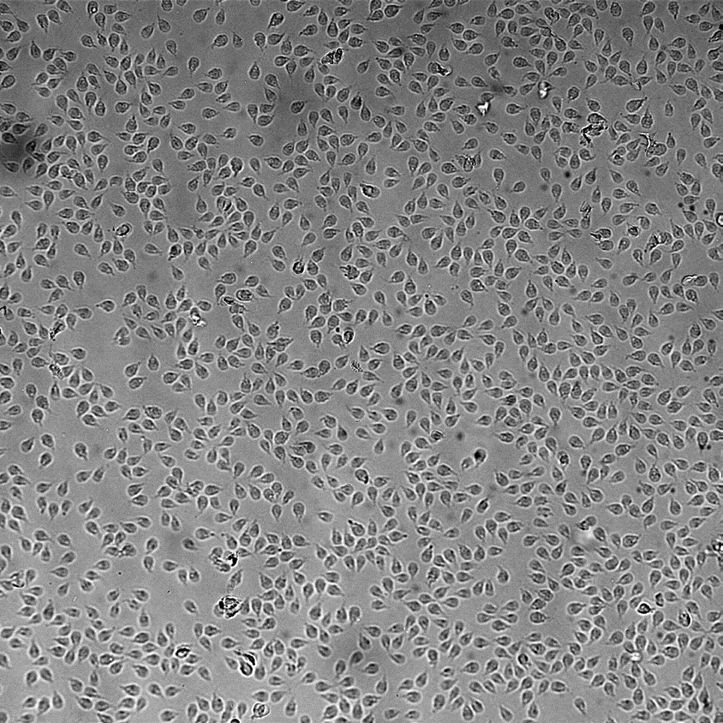


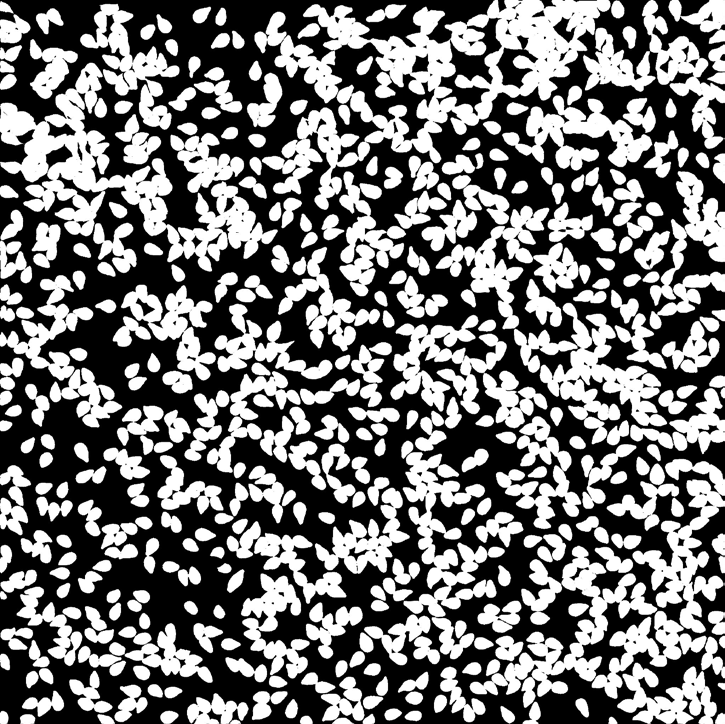


Thresholding depending on size

Number of cells with known area (each)

Calculating the percentage of the total image area occupied by all objects.

Averaging obtained occupancy per well

Occupancy per

image [%]

Occupancy per well [%]
